# Structure and Sensor Properties of Thin Ordered Solid Films

**DOI:** 10.3390/s91007733

**Published:** 2009-09-28

**Authors:** Jadwiga Sołoducho, Joanna Cabaj, Agnieszka Świst

**Affiliations:** Department of Medicinal Chemistry and Microbiology, Faculty of Chemistry, Wrocław University of Technology, Wybrzeże Wyspiańskiego 27, 50-370 Wrocław, Poland; E-Mails: joanna.cabaj@pwr.wroc.pl (J.C.); agnieszka.swist@pwr.wroc.pl (A.S.)

**Keywords:** Langmuir-Blodgett films, laccase, tyrosinase, diphenylamine and carbazole derivatives, biosensing effect, gas sensors, electroconductivity, AFM

## Abstract

Miniaturized gas sensors and biosensors based on nanostructured sensing elements have attracted considerable interest because these nanostructured materials can be used to significantly improve sensor sensitivity and the response time. We report here on a generic, reversible sensing platform based on hybrid nanofilms. Thin ordered Langmuir-Blodgett (LB) films built of fluorene derivatives were used as effective gas sensors for both oxidative and reductive analytes. A novel immobilization method based on thin LB films as a matrix has been developed for construction of sensing protein layers. Biomolecules can often be incorporated into and immobilized on Langmuir-Blodgett films using adsorption methods or by covalent immobilization of proteins. The sensor sensitisation was achieved by an amphiphilic *N*-alkyl-bis(thiophene)arylenes admixed into the film. The interlaced derivative was expected to facilitate the electron transfer, thereby enhancing the sensor sensitivity. The results suggest that this may be very promising approach for exploring the interactions between proteins and high throughput detection of phenol derivatives in wastewater.

## 1. Introduction

A variety of different chemical sensors based on conducting molecules can be used for gaseous or liquid analytes. Conductometric electronic noses based on conducting structures have been applied to the detection of odours connected with fires [1], aromatic hydrocarbons [2], pollutants in water [3,4] or for analysis of wine [5]. As shown in many articles, the unique properties of conducting structures can be used advantageously in various chemical and biological sensors, which can be constructed by modification of substrates with suitable conducting materials.

The use of conducting units for the detection of gaseous analytes belongs to the well developed field of chemosensor design. Gases interacting with conducting materials can be divided into two classes: gases which chemically react with a material and those that physically adsorb on it. Chemical reactions lead to changes in the doping levels of conducting materials and therefore alter their physical properties like resistance. Electron acceptors like NO_2_ and I_2_ are able to oxidize partially reduced conducting materials and therefore increase their doping levels. To oxidize conducting materials, the gases should have a higher electron affinity than the material. NO_2_ was found to increase the number of charge carriers in polyaniline [6] through oxidative doping with NO_2_^-^ions and therefore decrease the resistance. Electron donating gases like NH_3_ reduce and dedope conducting materials by electrochemical removal of the counterion, which leads to an increase in resistance. This process rarely goes to completion due to limitations arising from the slow diffusion of counterions from the solid polymer matrix. There is also a chemical compensation process in which the oxidized polymer (p-type doped) reacts with a reducing agent such as NH_3_ to regain the original insulating properties. The compensating reagent diffuses into the polymer matrix and neutralizes the electrical charge of the system by a chemical reaction involving electron transfer. Ammonia was found to decrease the conductivity of polythiophenes [7,8].

The design of affinity biosensor devices is based on immobilization of specific molecule systems, e.g., antibodies and natural protein receptors, connected to a transducer element. The essential features for the sensitivity of the system are an optimized surface density, good accessibility, long-term stability and minimal non-specific interactions with the analytes.

Biosensors generally offer simplified reagentless analyses for a range of biomedical and industrial applications. For any sensor, speed of response and reversibility are often paramount. In solid-state sensors, analyte molecules have to diffuse into the acting sensing component and any reaction product must diffuse out. It therefore follows that the thinner the sensing layer is, the shorter the response time is and thereby speed and reversibility are improved. Such a model of molecular assemblies can be prepared by Langmuir-Blodgett (LB) and Langmuir-Schaefer (LS) techniques or by using self-assembly monolayers. LB layers of amphiphile–protein complexes with embedded immobilized enzymes could be also deposited directly on transducers (such as amperometric or potentiometric electrodes or field effect transistors) and thus used as recognition elements [9].

Only very few enzymes or proteins can form sole LB films, but most protein molecules can be incorporated to a solid surface by adsorption from solutions and their subsequent binding to an aliphatic acid film [10] *via* a -COOH group or by covalent cross-linking (including in LB film). In some cases, immobilization of proteins on the solid substrate can improve the sensing stability and allow for their reuse (Table 1).

Among enzymes, laccases and tyrosinases are two groups of phenol oxidases that catalyze the transformation of a large number of phenolic and non-phenolic aromatic compounds. Abundant information is available in the literature on the use of free and immobilized phenol oxidases in several applied areas [18,19]. To date, however, an exhaustive overview in the basic aspects of immoblilization of laccase and tyrosinase has been lacking. To retain the enzymes' specific biological function, their immobilization on a solid matrix is a key factor in preparing biosensors. So far several immobilization strategies have been commonly used to immobilize small molecules onto appropriately functionalized glass slides, including covalent immobilization with Staudingeer ligation [20]. Immobilization methods for tyrosinase such as physical adsorption, covalent cross-linking, incorporation within carbon paste, immobilization in polymer films, entrapment in cyro-hydrogel and some sol–gel matrices have also been reported in the literature [17].

Conducting structures formed by deposition are of great interest as sensors. Suitable polymers can be incorporated into LB structures and for example, polyaniline/glucose oxidase LB film can be deposited and used as an electrochemical sensor for glucose with a linear response to 30 mM [21]. Other conducting polymers retain their electroactivity and detect glucose or urea [12,13].

**Scheme 1.**
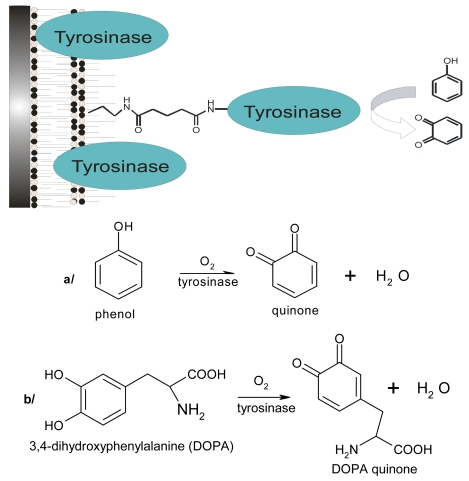
Catalytic effect of immobilized tyrosinase [17], oxidation of phenol (a) and 3,4-dihydroxyphenylalanine (b).

Here, we discuss chemical and biological sensors based on conducting materials. The topic is divided into sections taking into account different functions of the sensors, according to the measured analyte. Fluorene-based conjugated polymers have emerged as a very promising class of materials for use in electronic sensor devices because of their thermal stability, good solubility, and facile functionalization at the C-9 position of fluorene. In order to benefit from the effect of phenoloxidases a novel sensor based on catalytic protein effect was developed for phenol derivative determination (Scheme 1). Preliminary results on the sensing properties of laccase from *Cerrena unicolor* and tyrosinase from *Agaricus bisporus* immobilized in LB film were obtained. Laccase displays a wide substrate specificity for reducing reagents, catalyzing the oxidation of different phenols and aromatic diamines.

**Figure 1.**
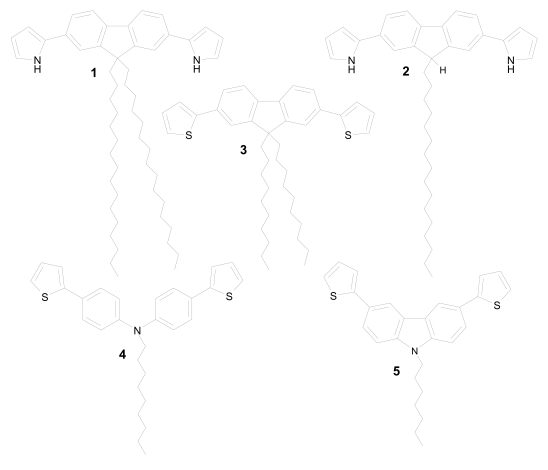
Some conducting structures used as chemical sensor elements: 9,9-dihexadecyl-2,7-bis(pyrrole-2-yl)fluorene (**1**), 9-hexadecyl-2,7-bis(pyrrole-2-yl)fluorene (**2**), 9,9-didecyl-2,7-bis(2-thiophene)fluorene (**3**), *N*-nonyl-4,4′-bis-(thiophene)diphenylamine (**4**), *N*-heptyl-3,6-bis(2-thiophene)carbazole (**5**).

In our sensors the enzyme immobilization was carried with a glutaraldehyde cross-linked protein film built of laccase or tyrosinase, *N*-nonyl-bis(thiophene)diphenylamine, *N*-heptyl-bis-(thiophene)-carbazole (Figure 1), and carboxylic acids (stearic acid, tricosenoic acid). The intercalated conducting units were expected to facilitate the electron transfer, enhancing the sensing properties.

## 2. Results and Discussion

### 2.1. Gas Sensor Devices

It has been known for some time now that either oxidizing or reducing adsorbed gases can substantially affect the electrical properties of polymers such as polythiophene or polypyrrole derivatives (Table 2) [22]. The changes in electrical conductivity can be exploited for a number of chemical or gas sensor applications, as known from literature, for example, in the case of a chemiresistor device built of dithiolene metal complexes [23] and an optical sensor for detection of gases such as nitrogen oxides built of a highly polarisable organic material (Polysiloxane I) [24]. These types of thin (100–200 nm) layer sensors give from three up to even 36 current responses in the presence of ammonia or HNO_3_ depending on the type of material employed (polyaniline, polythiophenes) [25,26].

A series of investigations of the electrical conductivity of Langmuir-Blodgett (LB) films built of one monolayer of 9,9-dihexadecyl-or 9-hexadecyl-2,7-bis(pyrrole-2-yl)fluorene (**1** and **2,** respectively [34]) and potential precursors of the new conducting polymers, exposed to different concentrations of gases or vapors (ethyl alcohol, ammonia and nitrogen dioxide) were carried out.

**Figure 2.**
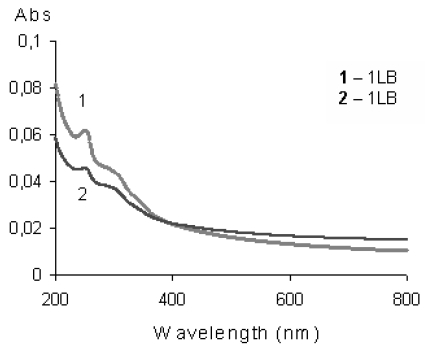
UV-Vis spectra of a one monolayer thick film of fluorene derivatives **1** and **2** [30].

The films were deposited onto a set of eight interdigital, buried Au electrodes photo-lithographically fixed on thermally SiO_2_-coated silicon substrates. This type of preparation of electrodes provides a flat, polished sensor surface ready to LB deposition [30]. Transfer of the monolayers of **1** and **2** to the substrate allowed preparation of complete and high quality Langmuir-Blodgett films, which was confirmed by the corresponding UV-Vis spectra (Figure 2).

The current-voltage characteristics were found to be linear for all gases of interest over the whole measurement range i.e., 0.1 to 4.0 V in an ambient atmosphere. The nominal concentrations of toxic gases ranged between 0.2 ppm to 6 ppm for NO_2_ and from 15 ppm to 886 ppm for NH_3._ The sample processing and electrical measurements, as well as preparation of gas mixtures were carried out at ca. 22 °C. The current flowing through as–deposited films in most cases ranged between 1 × 10^-5^A and 1.2 × 10^-7^A at room temperatures. An increase in current was observed after every new portion of gases (1.18 ppm-NO_2_, 15 ppm-NH_3_) added.

**Figure 3.**
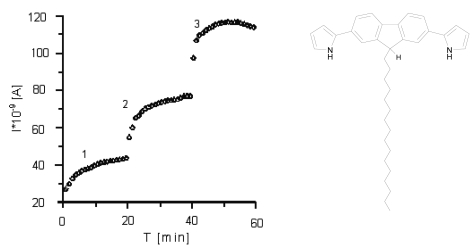
Response of a one LB layer thick sensor **2** to increasing concentrations of NO_2_ during 60 minutes. In each step, the NO_2_ concentration was increased by 1.18 ppm. U = 0.5 V, the initial signal as a red point [30].

Upon exposure to NO_2_ the conductivity of a pristine film of monosubstituted derivative **2** increased substantially. The first exposure to NO_2_ gas causes a conductivity growth dependent on the NO_2_ concentration, exhibiting a rather strong tendency to saturation at higher gas concentrations (first step, Figure 3). After the complete series of measurements, the measuring box was opened to the air and recovery of conductivity was observed after *ca*. 20 minutes. The second and all consecutive admissions of gases resulted in a similar dependence of conductivity of NO_2_ concentrations, but with a higher sensor sensitivity (second and third steps, Figure 3). The subsequent exposure to air resulted in a decrease in the conductivity to almost the initial value. The effect was found to be reversible in all cases.

**Figure 4.**
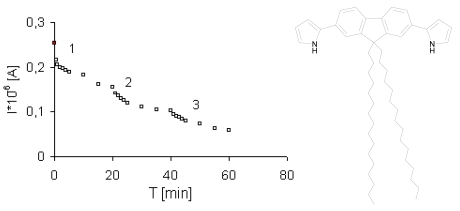
Response of a one LB layer thick sensor **1** to increasing concentrations of NH_3_ during 60 minutes. In each step, the NH_3_ concentration was increased by 15 ppm. U = 0.5 V, the initial signal as red point [30].

**Figure 5.**
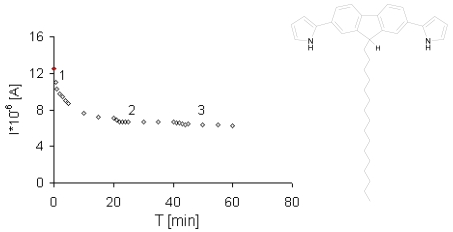
Response of one LB layer thick sensor **2** to increasing concentrations of NH_3_ during 60 minutes. In each step, the NH_3_ concentration was elevated by 15 ppm. U = 0.4 V, the initial signal as red point [30].

Similar experiments were also performed for ammonia and ethanol. However, the electrical conductivity of the film decreased with increases of the NH_3_ and ethanol concentrations (Figures 4 and 5). Electron donating gases like NH_3_ electrochemically remove the counterion from conducting materials, what leads to an increase in resistance.

The electon–acceptor character of a pyrrole ring in the structures **1** and **2** causes an increase in electrical conductivity in the presence of oxidative gases, and a decrease of conductivity in the presence of reducing ones, both circumstances were observed. Moreover, the high volatility of ammonia allows for complete desorption from the surface during the regeneration process.

Results of analogous measurements performed for dialkylsubstituted fluorene derivative **1** were similar to the findings for the LB film made of compound **1**. Details of the sensor measurement methodology were published by us previously [35]. Relatively short response times and fair sensitivity at room temperatures and good reproducibility of the results make these materials very promising candidates for gas sensing elements.

When a molecule of an oxidizing or reducing gas is chemisorbed on the surface of a semiconductor, a charge transfer may occur between them. It depends upon the electronegativity of the gas and the work function of the polymer or solid. The charge transfer can influence not only the surface conductivity of the semiconductor but also, the subsequent reaction on the surface, charges can be injected into the bulk causing changes in both the surface and bulk conductivities. If one assumes that the conductivity is directly related to the number of molecules of the active gas adsorbed on the sample surface, then under a constant voltage, the current as a function or partial pressure of the gas can be described by the Frendlich isotherm [30].

#### 2.1.1. Conductivity of LB Films Built of Fluorene Charge Transfer Complex

The charge transfer (CT) complexes of 7,7,8,8-tetracyanoquinodimethane (TCNQ) have anisotropic structural, electrical, optical, and magnetic properties. These complexes offer alternative possibilities for semiconductive materials with wide technological applications [36]. The structural and electronic properties of the complex are of interest because they are thought to be important determinants of electron-transport properties. The CT complex of a fluorene derivative with tetracyanoquinodimethane (TCNQ) was prepared by refluxing an equimolar solution of 9,9-didecyl-2,7-bis(2-thiophene)fluorene (**3**) and TCNQ in chloroform or acetonitrile, until the reaction was completed [37]. Then organic layers were deposited by LB technique according to standard methods.

The preparation of gas mixtures, sample processing and all conductivity measurements were carried out as usual at *ca*. 22 °C. The current-voltage characteristics were measured, prior to the admission of the gas and under different partial pressures of NO_2_, and were found linear over the entire voltage range covered by the experiment.

**Figure 6.**
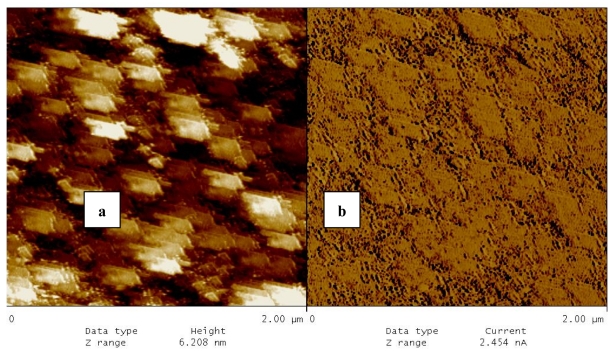
AFM photomicrographs of **3**–TCNQ complex LB film deposited on ITO substrates; (a) real pictures of surface topography [37].

The transference of the LB film was Y–type on first deposition and Z–type in following ones. At first, the LB film quality of **3**–TCNQ complex deposited on an indium thin oxide (ITO)-coated substrate was investigated (Figure 6). The atomic force microscopy (AFM) measurements revealed that for homogenous films, even with great magnifications, the heap-like texture of LB films was clearly observed. The main characteristic of the film is that all of the heaps have more or less the same size (film grain size of ∼250 nm). The film roughness was measured as 24.3 nm. The topographies of layers indicate that the CT complex was fairly well deposited onto the surface.

**Figure 7.**
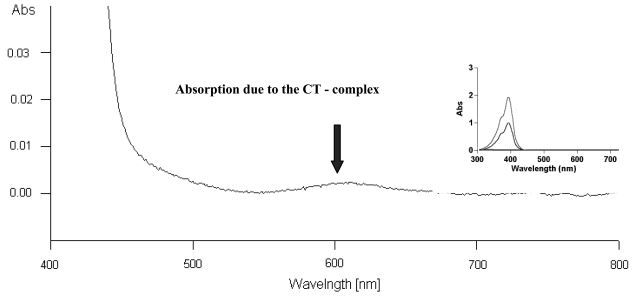
Absorption spectrum of the **3**–TCNQ complex in a LB film (3 layers) [37], absorption spectrum of TCNQ as an inset.

It seems then that the presence of TCNQ changes the hydrophilic/hydrophobic character of the film surface. The relationship between absorbance and the number of layers and the constant transfer ratio during the deposition indicated on constant architecture of LB film layers. The absorption spectrum of **3**–TCNQ complex deposited film confirms the formation of CT-complex. Figure 7 shows a characteristic CT band observed at 600 nm, while there was no significant absorption in the pristine TCNQ solution (inset in Figure 7, max 394 nm). We found a broad TCNQ complex absorption that is connected with the solid state of TCNQ derivatives. In solid state they become broader and are shifted toward longer wavelengths.

**Figure 8.**
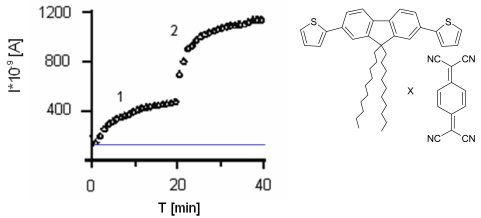
Conductivity response of a five layer LB film of **3**-TCNQ in the presence of NO_2._ The first and second admission of the gas, baseline signal-blue line.

**Figure 9.**
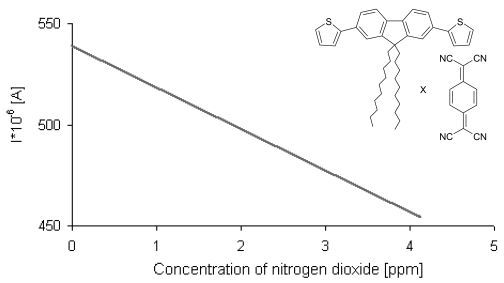
Conductivity response of a five layer LB film to increasing NO_2_ concentrations in the atmosphere surrounding the sample; U = 15 mV [37].

The investigations were focused on measuring the dependence of the surface conductivity of LB films built of **3**–TCNQ CT complex sensors exposed to air at ambient pressure, containing NO_2_, against the concentration of the gas. The nominal concentration of gas ranged between 1–5 ppm of NO_2_. During the first gas admission a linear dependence of conductivity on the gas concentration was observed (Figures 8 and 9). After the series of measurements, the measuring box was opened to the air and recovery of conductivity was observed after half an hour.

The second and all consecutive admissions of gas resulted in the same character of the dependence of conductivity on the gas concentration (Figure 9).

In all measuring cycles the response time of the sensor was in the range of seconds and almost full recovery, at room temperature, was achieved within 20 minutes or less, after the measuring chamber was opened. No heating was necessary for desorption of gas from the surface of the sensor. Relatively short response times and fair sensitivity at room temperatures make these materials very promising candidates for gas sensing elements.

In the case of CT complexes both types of gases (oxidative and reductive) engender noticeable changes of conductivity. The electronic conduction in organic molecular-based compound of the TCNQ family arises from a charge transfer between the constituent acceptor (TCNQ) and donor molecules (9,9-dialkylfluorene derivatives) [37].

### 2.2. Enzymatic Sensor Devices

A wide range of materials have been electrodeposited onto electrode surfaces (polyaniline, polyphenol, polythiophene [10]) and used to immobilize biomolecules. These films tend to be relatively thick. Much research has been performed over many years on the fabrication of thin films of wide variety of materials. Incorporation of biologically active materials into these films leads to the construction of biosensors.

**Figure 10.**
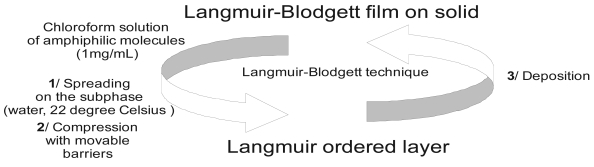
Simplified scheme of preparation LB films.

One of the procedures that allows one to obtain one molecule thick layers is the Langmuir-Blodgett technique. Therefore, the ability to control the LB deposition enables well-ordered thin amphiphilic films as matrixes for immobilization of proteins. Conditions for the preparation and transfer of the LB films and compositions of the films with and without diphenylamine derivative (**4**, Scheme 1) are listed in Figure 10 and Table 3 (films **a**, **b**, **c**, **d**).

The amphiphilic **4** or **5**, fatty acids and phenoloxidases were dissolved in chloroform and mixed in equimolar proportions. Concentration of each solution was maintained at ca. 1 mg mL^-1^. The mixture was spread on a water subphase and the monolayer was compressed with movable barriers at a rate of 50 mm min^-1^. The deposition was Y-type with a transfer ratio of very close to unity, and the π-A isotherms were recorded by means of a commercial LB trough (KSV, System 5000).

For a covalent cross-linking of laccase on the modified surface an obtained LB film was sprinkled with one millilitre of glutaraldehyde (GA). In each case, immediately after applying the protein to thin LB layers, the substrates were placed in a desiccator. The process of immobilization was carried out for 12 hours, at 4 °C in a humid environment.

**Figure 11.**
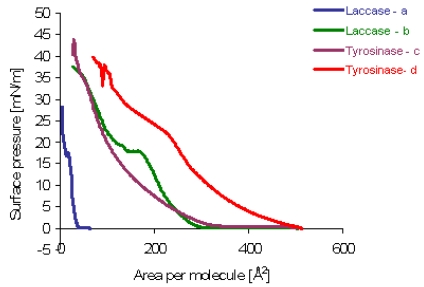
Surface pressure–area isotherms of amphiphilic diphenylamine derivative **4** incorporated film **b, c** and **4** free film **a, d** at 22 °C on pure water [17].

The components listed in Table 3 are visible in Figure 11. The clear liquid–expanded to liquid–condensed phase transition (LE-LC) is visible in the isotherms of mixed monolayers. However, the structure of the condensed region is rather poorly resolved from the isotherms.

As previously shown [38], the inverse isothermal compressibility coefficient 1/kT can be used to obtain more detailed information about the phase transitions during compression. The two-dimensional 1/kT depends on the state of the film and is defined as follows [39]:

1/kT=(δπ/δA)T

where 1/kT is shown as a function of mean molecular area for the binary monolayer of two different proteins, **b** (laccase) and **c** (tyrosinase). The LE phase is typical with the values of 1/kT ranging near 50 mN/m (**b**–55 mN/m, **c**–40.8 mN/m) [17]. The condensed phases in turn, appear with smaller molecular areas, with values of 1/kT close to 100 mN/m (**b**–97 mN/m, **c**–107 mN/m) [17].

Two local maxima in the LC region observed for isotherms **c** and **d** are interpreted to represent two distinct condensed phases, the liquid condensed (LCo) and more rigid liquid crystalline (LCr) phases (this phase is able to crystallize), indicated by the absolute values of 1/kT. LC phases were more clearly separated and also appeared at smaller mean molecular areas. However, neither **c** nor **d** monolayers reached the solid crystalline ordered state because the values of 1/kT remained smaller than the values characteristic for this state (1/kT > 1,000 mN/m) [39].

#### 2.2.1. Detection of Biosensing Effect of Fabricated LB Protein Films

**Figure 12.**
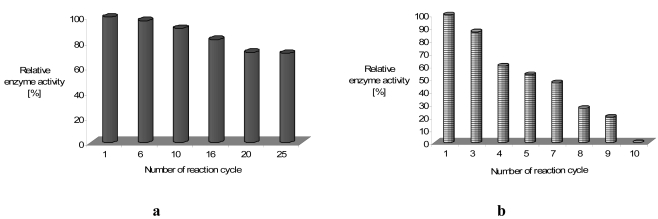
Immobilized laccase-**a**, and tyrosinase-**b** activity during repeated reaction cycle in presence of 2,2′-azino-bis(3-ethylbenzthiazoline-6-sulphonic acid)-ABTS as substrate (0.228 mM) [17].

Since the immobilization of laccase on LB films was achieved through the cross-linking reaction with glutaraldehyde, its amount reflects the immobilized enzyme activity. In our case, laccase incorporated into obtained film had an initial enzyme activity of merely 10% of the activity of the native laccase. In case of tyrosinase an initial protein activity was close to about 3.5% of free protein [17]. As one can see from Figure 12, the sensing activity of the phenoloxidases cross-linked into LB films *via* glutaraldehyde, is rather stable and reproducible, especially for the laccase sensor. The sensing activity of the laccase, covalently immobilized onto LB films was reproducible during up to 25 incubation cycles (repeated reaction of oxidizing reagent catalyzed by immobilized laccase). The observed decrease of enzyme activity is rather small and the cross-linked protein is active for a few months.

ABTS as a standard enzyme activity indicator used for the reaction catalyzed by laccase showed much higher protein activity compared to the natural reagents like *o*-aminophenol or catechol. In the case of natural reagents (Table 4) the laccase activity in the film, although is as low as 40-1% of the activity of laccase in presence of 2,2′-azino-bis(3-ethylbenzthiazoline-6-sulphonic acid)-ABTS, is stable and reproducible [17].

The surface of every molecule in the mixed LB film (calculated from area per molecule) suggests that diphenylamine or bis(thiophene)carbazole molecules **4** and **5** are squeezed from the carboxylic heads of stearic/tricosenoic acid layer with their aliphatic chains being parallel. It also allows glutaraldehyde to slip into the created channels (pockets) and interact, *via* its aldehyde group with the divalent sulphur of thiophene, while the other aldehyde groups of glutaraldehyde are exposed to react with the lysine groups of the laccase or tyosinase. This “multilysinic” bonding of enzymes with the LB films opens in some respects the access to its active centres. Moreover, the grafting of enzyme into LB layers can provide better electrosterical stabilisation, due to the high molecular weight of the protein and the numerous functional groups in the protein structure (carboxyl, amine, etc.) that are potential charge carriers [40,41].

#### 2.2.2. Conjugated Mediators

**Figure 13.**
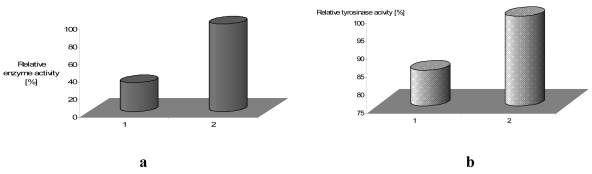
Activity of laccase –**a** and tyrosinase-**b** immobilized in LB film, 1-without **4** as mediator, 2-with **4**, substrate-ABTS (0.228 mM).

If the additional bis(thiophene)diphenylamine (**4**) or bis(thiophene)carbazole (**5**) molecules, acting as an electron mediator are present in the system they significantly enhance the mediating efficiency of reagents. In Figure 13 we have shown the effect of equimolar addition of **4** to protein; enzyme activity increased almost three-fold in the case of laccase (for tyrosinase we have found the increase in the activity by 15%, and it retained ca. 70% of its initial activity for as long as four months).

In general, a mediator could be a sort of ‘electron shuttle’ that, after being oxidised by the enzyme, diffuses away from the active site to oxidise any substrate that, because of its size, could not enter the enzymatic pocket directly. In addition, the oxidised form of the mediator being structurally ‘diverse’ from the enzyme, thereby extending the range of substrates susceptible to the enzymatic action [42,43]. Suitable enzyme–mediator systems could also enable the environmentally benign. Understanding the role and mechanism of action of these mediators is a practical issue. ABTS is the most common mediator for laccase activity and not the most efficient one. As could be seen from enzyme activity in Figure 13, the presence of additional molecules with conjugated bonds (**4**) in the system improves ABTS as well as *tert*-butylcatechol mediating efficiency significantly.

#### 2.2.3. AFM Study of Langmuir-Blodgett Films

**Figure 14.**
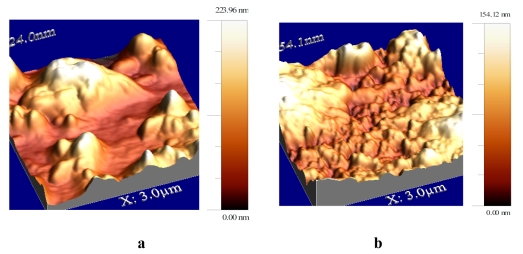
AFM images of stearic acid/**4**/laccase-**a** and stearic acid/**4**/tyrosinase LB film-**b**. All images are 3 μm × 3 μm [17].

Atomic force microscopy was used for the topographic characterization of the laccase and tyrosinase LB films deposited on quartz microscope slides (Figures 14 and 15). Cross-linked proteins were observed as characteristic islands (aggregates, grain size 10–200 nm). The roughness of stearic acid/**4**/laccase was measured to be 38.2 nm while the roughness value of tyrosinase film was found as 24.5 nm, similar results was found for film of lipase [44], whereas the roughness of glucose oxidase LB film has been measured as 0.38 nm [45]. The topographies of protein G LB films indicate that the protein G molecules were fairly well deposited onto the Au surface. But protein molecules were also adsorbed onto the Au substrate as an aggregated pattern in solid-like state with keeping its random cloud-like structure [46]. These obtained values were attributed to the immobilization process of comparatively large molecule aggregates of enzyme (laccase, tyrosinase) incorporated of the LB film. This leads to the conclusion that there is formation of an agglomerate of enzymes rather than an organized monolayer at the air/aqueous interface. The AFM results showed that the effect could be also associated with changes in the enzyme conformations. A monolayer rearrangement, such as two-dimensional formation or hindered molecular orientation, might take place during the phase transition behavior resulting in the molecular aggregates on the protein layer.

## 4. Conclusions

For nearly 50 years we have witnessed tremendous progress in the development of chemical and biological sensors. Elegant research on new sensing concepts, coupled with numerous technological innovations, has thus opened the perspective to applications of sensors and biosensors. Using modified or unmodified conducting structures as a receptor material or as one of components of the receptor layer in chemical sensors offer a wide range of applications as one of the most stable detection layers.

It is shown that the layers built of fluorene derivatives successfully deposited as LB films, may be used as gas sensors responding to nitrogen dioxide, ethanol and ammonia. It is important to note that these films can be recovered without any thermal treatment–just by exposure to air.

Different responses to different gases at various concentrations obtained for mono-and disubstituted derivatives of bis(arylene)fluorene allow to think that they can be used as elements of gas sensing devices of the type of an “artificial nose” or a neuronic network.

Furthermore, enzymes immobilized in thin films constitute nearly 85% of the world market for biosensors. Major fundamental and technological advances have been made for also enhancing the capabilities and improving the reliability of phenol measuring devices. The success of wastewater meters had stimulated considerable interest in devices for monitoring important compounds. Similarly, new materials (matrixes, mediators, etc.) and concepts, developed originally, now benefit a wide range of sensing applications.

In this study, a sensor layer was presented in order to investigate the mediator effect of diphenylamine or carbazole derivatives on the activity of immobilized laccase and tyrosinase. A heterogeneous LB film, consisting of amphiphilic bis(thiophene)arylene and long-chain carboxylic acid modified by glutaraldehyde provides sites for successful phenolaxidase immobilization. By using the biosensors we detected a linear concentration range of oxidized reagents. Enzyme immobilized by this technique is active and stable for at least three months.

The fact of sensitization of sensing system with presence of mediating conjugated amphiphile can be recognized as a successful step into enhancing a biosensor activities, leading perhaps to manufacturing differ protein electrodes by use conjugated, appropriate amphiphilic mediators.

A reproducibility of the sensing effects of the biosensor is constructed with enzymes immobilized in LB film and interactions with mediated bis(thiophene)arylene derivatives is subject of our further investigations and could be alternative method for routine analysis of wastewater.

As this field enters its fifth decade of intense research, we expect significant efforts that couple the fundamental sciences with technological advances. This stretching of the ingenuity of researches will result in advances including the use of nanomaterials for improved also electrical contact between the redox centre and electrode supports.

## Figures and Tables

**Table 1.** Comparison of selected protein sensors immobilized in thin films.

***Immobilization method***	***Sample thickness***	***Response time***	***Stability***	***Reference***
Glucose oxidase LB deposition with lipids	2 layers	0.12 min	3 month	[11]
Glucose oxidase LB deposition with polythiophene	1 layer	2 min	40 days	[12,13]
Glucose oxidase LB deposition with C_18_H_37_N^+^Me_3_	1 layer	not reported	not reported	[14]
Laccase LB deposition with *N*-heptyl-bis(thiophene)carbazole and tricosenoic acid cross-linked with glutaraldehyde	5 layers	1.5 min	>3 month	[15]
Horseradish peroxidase LB deposition with phospholipids	1 layer	not reported	>2 weeks	[16]
Laccase LB deposition with *N*-nonyl-bis(thiophene)diphenylamine and stearic acid cross-linked with glutaraldehyde	5 layers	1.5 min	>3 month	[17]
Tyrosinase LB deposition with *N*-nonyl-bis(thiophene)diphenylamine and stearic acid cross-linked with glutaraldehyde	5 layers	2 min	>3 month	[17]

**Table 2.** Selected few examples of gaseous and vapor sensors based on conducting structures.

***Analyte***	***Polymer or monomer***	***Transduction***	***References***
HCl	Polyaniline	Conductometric	[25,27]
NH_3_	Polyaniline, polypyrrole, LB film of 9,9-dihexadecyl-2,7-bis(pyrrole-2-yl)fluorene	Conductometric	[28-30],

NO_2_	Polyaniline, poly(3-hexylthiophene), LB film of 9-hexadecyl-2,7-bis(pyrrole-2-yl)fluorene	Conductometric	[30-32]
NH_4_^+^	Polypyrrole	Amperometric	[33]
EtOH	LB film of 9,9-dihexadecyl-2,7-bis(pyrrole-2-yl)fluorene	Amperometric	[30]

**Table 3.** Compositions of Langmuir-Blodgett films and their transfer conditions [15,17]

***Film***	***Enzyme***	***Carbazole derivative (5)***	***Diphenylamine derivative (4)***	***Stearic or tricosenoic acid***	***Transfer pressure (mN m^-1^)***	***Temperature of transfer (°C)***
**1.**	Laccase	1		1	25	22
**2.**	Laccase-**a**			1	25	22
**3.**	Laccase-**b**		1	1	25	22
**4.**	Tyrosinase-**c**		1	1	27	22
**5.**	Tyrosinase-**d**			1	27	22

**Table 4.** Immobilized proteins activity in presence of various phenolic compounds [17].

***Enzyme***	***Reagent***	***Relative enzyme activity [%]***
Laccase	ABTS, 0.228mM	100
Laccase	Phenol, 10 mM	0.98
Laccase	Catechol, 10 mM	12.5
Laccase	*o*-Aminophenol, 10 mM	38.5
**Tyrosinase**	*tert*-Butylcatechol, 5 mM	18.2
